# Excitation Wavelength Engineering through Organic Linker Choice in Luminescent Atomic/Molecular Layer Deposited Lanthanide–Organic Thin Films

**DOI:** 10.1021/acs.chemmater.3c00955

**Published:** 2023-07-17

**Authors:** Amr Ghazy, Mika Lastusaari, Maarit Karppinen

**Affiliations:** † Department of Chemistry and Materials Science, Aalto University, Espoo FI-00076, Finland; ‡ Department of Chemistry, University of Turku, Turku FI-20014, Finland

## Abstract

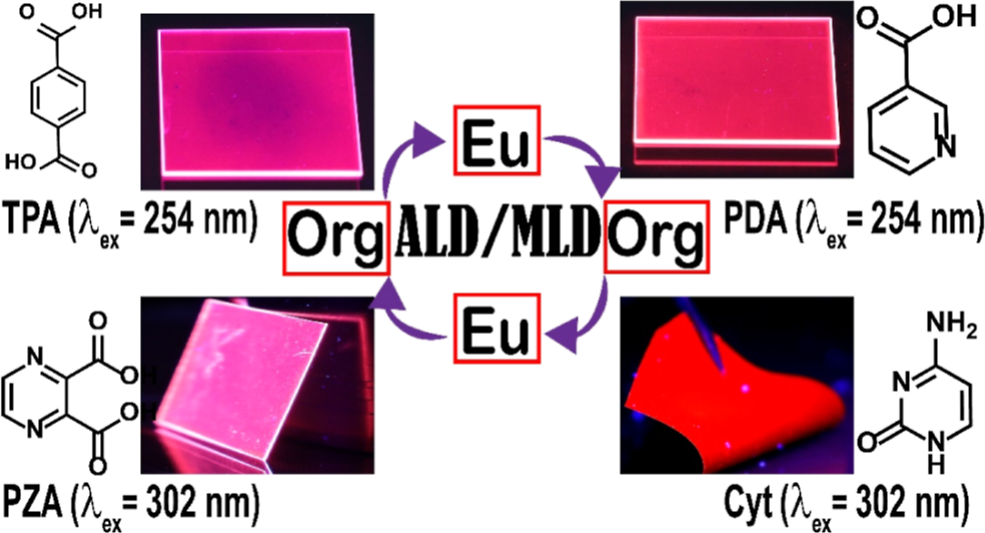

We demonstrate multiple roles for the organic linker in luminescent lanthanide–organic thin films grown with the strongly emerging atomic/molecular layer deposition technique. Besides rendering the hybrid thin film mechanically flexible and keeping the lanthanide nodes at a distance adequate to avoid concentration quenching, the organic moieties can act as efficient sensitizers for the lanthanide luminescence. We investigate six different aromatic organic precursors in combination with Eu^3+^ ions to reveal that by introducing different nitrogen species within the aromatic ring, it is possible to extend the excitation wavelength area from the UV range to the visible range. This opens new horizons for the application space of these efficiently photoluminescent thin-film materials.

## 1 Introduction

Trivalent lanthanide (Ln) ions possess attractive luminescence characteristics relevant to various applications in displays, lighting, imaging, and sensing owing to their sharp 4f–4f emission peaks located over a wide range of wavelengths from ultraviolet (UV) to visible and even near-infrared (IR) areas depending on the Ln^3+^ species.^1^ The spatially extended 5s and 5p orbitals offer shielding to the 4f electrons, which makes the 4f–4f emission independent of the host lattice or the ligand environment. This enables narrow emission bands, long luminescence lifetime, high color purity, and high color rendering index.^2,3^

The downside is that the 4f–4f transitions of Ln^3+^ ions are parity forbidden, meaning that the absorption coefficients are low, and the direct excitation is inefficient. A possible route to enhance the luminescence efficiency is to combine the Ln^3+^ ions with organic π-conjugated aromatic ligands, capable of absorbing UV light and transferring the absorbed energy to the Ln^3+^ ions via a so-called antenna effect.^4^ Hence, lanthanide–organic complexes have attracted considerable attention as viable luminescence materials,^5^ and in particular, Tb^3+^, Eu^3+^, and Sm^3+^ complexes have been investigated for their characteristic monochromatic green, red, and deep red emissions, respectively, for displays, lighting, and sensing devices.^6−10^ Similarly, the near-IR emissions of Nd^3+^, Er^3+^, and Yb^3+^ complexes with long emission lifetimes could be utilized in bioimaging applications;^11^ also, efficient wavelength converters using lanthanide complexes for silicon solar cells have been reported.^12^ The nature of the organic ligand also plays a role,^13^ and for example, nitrogen-containing molecules have been highlighted.^14^

Modern applications of luminescence materials based on increasingly complex/nanoscale device structures call for new material design and fabrication approaches, especially to produce these materials as high-quality thin films or conformal coatings. Conventionally, lanthanide–organic thin films have been produced through solvothermal routes, such as spin coating or freeze drying.^15,16^ However, these techniques lack precise control over the film thickness and conformality; moreover, they tend to leave traces of solvents in the targeted functional coating. In recent years, atomic/molecular layer deposition (ALD/MLD) has been strongly emerging as a state-of-the-art fabrication route for high-quality metal–organic thin films.^17,18^ The parent ALD technique has been the fastest growing thin film technology in microelectronics already for decades,^19^ while the MLD counterpart for organic thin films was invented much later.^20^ Both techniques are similar in that they are based on sequential pulsing of two (or more) different and mutually reactive gas-phase precursors and have the capacity to yield precisely thickness-controlled and large-area homogeneous thin films even on complex substrate architectures.^21^ The modularity of the two techniques then allows their combination into the desired hybrid metal–organic compositions.^22,23^ Importantly, the mechanical properties of ALD/MLD-grown hybrid films are superior to those of purely inorganic thin films^24^

The ALD fabrication of purely inorganic Ln_2_O_3_ thin films is well established; these films are typically grown from Ln(thd)_3_ (thd = 2,2,6,6-tetramethyl-3,5-heptanedione) using ozone as the co-reactant at relatively high temperatures (ca. 300 °C).^25,26^ However, these oxide films based on a single Ln species suffer from the concentration-quenching effect and are not luminescent.^27,28^ The concentration quenching issue is typically avoided in bulk inorganic materials by mixing the luminescent Ln species with non-luminescent Ln species for appropriate dilution.^29,30^ This could be realized in ALD too, but it would lead to a more complex three-precursor process. Moreover, the system would still suffer from the small absorption cross-section and ineffective direct excitation issues; hence, including the organic linker is highly beneficial.^31^ Indeed, in the ALD/MLD-grown Ln–organic films, the organic linkers provide the required separation between the emitting Ln^3+^ ions to overcome the concentration quenching issue; already, various luminescent Ln–organic thin films have been grown with ALD/MLD.^31−33^ In these films, terephthalic acid (TPA)(benzene-1,4-dicarboxylic acid) has been the most commonly employed organic precursor,^32,34^ owing to its relatively low sublimation temperature (185 °C) and appreciably high reactivity toward the Ln(thd)_3_ precursors.^35^ While the Ln–TPA films offer strong emission intensity, their narrow excitation bands are located in the short wavelength area around 250 nm, thus restricting the potential applications to UV-excited systems only.^34,36−38^

**Figure 1:**
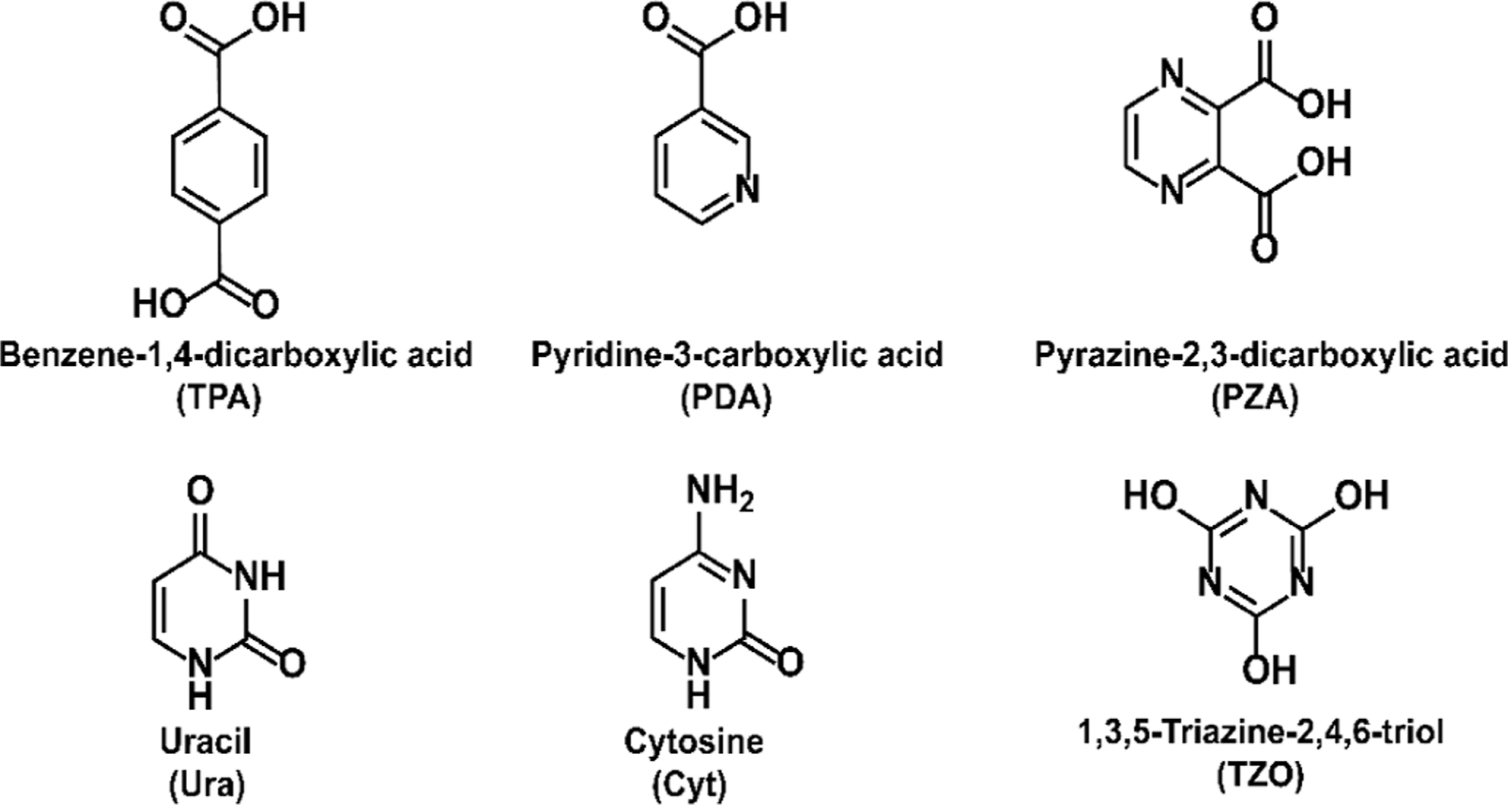
Organic precursors employed in this work: molecular structure, name, and abbreviation used.

In this contribution, we will demonstrate that nitrogen atoms within the aromatic ring of the organic linker molecule may significantly extend the absorption range of the organic moiety in the Ln–organic thin films toward the higher wavelengths, such that the Ln^3+^ luminescence can be realized even with visible light excitation. We investigate five different organic precursors with N-containing aromatic rings; Figure 1 shows the molecular structures of these organic precursors.

For the lanthanide species, europium was selected owing to its well-known intense red luminescence. Half of the organic precursors investigated are carboxylic acids, which are known to react well with common metal-bearing precursors such as Ln(thd)_3_. Pyridine-3-carboxylic acid (PDA) (also known as nicotinic acid) has one nitrogen atom within the aromatic ring, while 2,3-pyrazine-dicarboxylic acid (PZA) has two nitrogen atoms within the ring. Additionally, we investigate the precursors with oxo (=O) or N-containing (−NH, −NH_2_) reactive groups. The two naturally occurring nucleobases, uracil (Ura) and cytosine (Cyt), both contain the pyrimidine ring, but differ from each other with respect to the reactive groups: Ura has two oxo groups, while Cyt has only one oxo group in combination with an amino group (−NH_2_). Finally, 1,3,5-triazine-2,4,6-triol [(TZO) also known as cyanuric acid] has a so-called triazine ring constituted of three nitrogen atoms and three carbons and additionally three −OH substituents as reactive groups. It is worth mentioning that TZO exists in two tautomer forms, triol or trione.

## 2 Experimental Section

All the Eu–organic films were deposited through a single-step ALD/MLD process in a flow-type hot-wall ALD reactor (F-120 by ASM Microchemistry Ltd.). The reactor pressure was maintained between 2 and 4 mbar, and in-house generated nitrogen was used both as the purging and carrier gas for all the processes. Samples were mostly deposited on Si(100) substrates, the size of which varied from ca. 10 × 10 mm^2^ to 25 × 30 mm^2^. The samples for absorption measurements were deposited on quartz glass substrates of 1 mm thickness and with a size of 30 × 30 mm^2^ (Finnish special glass). Additional samples were deposited on thin elastic glass with a 0.1 mm thickness and a 30 × 30 mm^2^ size (Schott AF32 eco) to demonstrate the mechanical flexibility of the films. All substrates were used as purchased with no further treatments.

As precursors, we employed Eu(thd)_3_ (thd = 2,2,6,6-tetramethyl-3,5-heptanedione) synthesized in-house as first described by Eisentraut and Sievers^39^ and commercially purchased organic precursors (PDA 99% , PZA 97%, Ura 99%, TZO 98% from Sigma-Aldrich; TPA 99%, and Cyt 98% from Tokyo chemical industries). All the precursors were solid powders; they were inserted into the reactor in open glass crucibles, which were heated for precursor sublimation during the depositions. The precursor heating temperatures and pulse/purge times are given in Table 1, together with the deposition temperatures applied. The TPA-, PDA-, and PZA-based processes were conducted at 180–190 °C (to avoid the melting of these carboxylic acid precursors), while for the rest of the processes (with Ura, Cyt, and TZO), the deposition temperature was 250 °C (as the non-acid organics are typically less reactive toward the metal-thd precursors). In each case, the number of deposition cycles was set such that the resultant film thickness was close to 50 nm, for the sake of better comparison.

**Table 1:** Deposition Parameters Applied and Resultant Basic Film Growth Characteristics: Precursor Heating Temperature (*T*_sub_), Deposition Temperature (*T*_dep_), Precursor Pulsing and Purging Times, and Growth Per Cycle (GPC), Density, and Roughness of ca. 50 nm Thin Films Deposited on Silicon

precursor	*T*_sub_ (°C)	*T*_dep_ (°C)	pulse (s)	purge (s)	GPC (Å/cycle)	density (g/cm^3^)	roughness (nm)
Eu(thd)_3_	140		3	4			
TPA	185	190	4	6	3.2	2.57	0.10
PDA	145	180	4	6	1.7	1.58	0.33
PZA	145	180	4	6	3.5	2.30	0.25
Ura	210	250	4	6	2.1	2.69	0.46
Cyt	210	250	4	6	1.5	2.22	0.15
TZO	200	250	4	6	2.1	3.56	0.98

The film thicknesses were determined using X-ray reflectivity (XRR) (X’Pert Pro MPD, PANalytical; Cu Kα). The XRR patterns were analyzed using X’Pert reflectivity software fitting to obtain, besides the film thickness, also the film density and roughness values. From standard X-ray diffraction experiments in preliminary experiments, all the films were found to be amorphous. Bonding structures were studied by Fourier transform infrared (FTIR) spectroscopy (Bruker Alpha II) performed in transmission mode in the range of 400–4000 cm^–1^. UV–vis absorption spectra were recorded via direct absorbance measurement (Shimadzu UV-2600), for the thin films grown on quartz glass.

Photoluminescence measurements were conducted (Edinburgh Instruments FLS1000) using a continuous-wave 450 W Xe lamp (Xe2) as the excitation source and PMT-900 photomultiplier tube as the detector. The emission spectra presented here were recorded using 250 nm as the excitation wavelength. All excitation spectra were recorded with a fixed emission wavelength of 615 nm, whereas the emission spectra were recorded at different excitation wavelengths between 250 and 350 nm.

## 3 Results and Discussion

### 3.1 Novel ALD/MLD Processes

All six ALD/MLD processes investigated were found to yield visibly homogeneous Eu–organic thin films on all the substrate types employed. It should be emphasized that among these processes, the Eu–TPA and Eu–PZA processes have been reported earlier,^37,40^ but the rest of the processes were newly developed here. However, Ura has been previously challenged as an organic precursor in ALD/MLD in combination with Na, Ba, La, and Ti.^34,41−43^ Hence, among the six organic precursors, PDA, Cyt, and TZO were challenged in the context of ALD/MLD for the first time in this work. Table 1 summarizes the film growth rates (expressed as so-called GPC or growth-per-cycle values), as well as the density and roughness values, determined for the films from the XRR data.

From Table 1, the two dicarboxylic acid (TPA and PZA)-based processes seem to progress with higher growth rates (GPC >3 Å/cycle) than the rest of the processes (GPC values between 1.5 and 2.1 Å/cycle). These higher GPC values are in line with the general understanding that carboxylic acid precursors are highly reactive toward metal-thd precursors. Here, it is worth noting that apparently two reactive carboxylic acid groups are needed to assure the high GPC, viz. the relatively low GPC value of 1.7 Å/cycle for the PDA precursor with only one −COOH group. In the investigated ALD/MLD processes—besides the carboxylic acid groups—other reactive groups presumably contribute to the film growth. This question was addressed through a detailed FTIR spectroscopy analysis, as discussed next. From the FTIR data, the different bonding sites/schemes between the Eu^3+^ ions and the organic linker molecules can be deduced. Importantly, the bonding scheme should be directly reflected in the packing/orientation of the organic linker molecules between the Eu^3+^ ions and thereby in the resultant film density. From Table 1, it is thus worth noting that the Eu–TZO film is significantly denser (3.56 g/cm^3^), and the Eu–PDA film is significantly less dense (1.58 g/cm^3^), than the rest of the films (densities between 2.2 and 2.7 g/cm^3^).

### 3.2 Chemical Bonding Schemes

**Figure 2:**
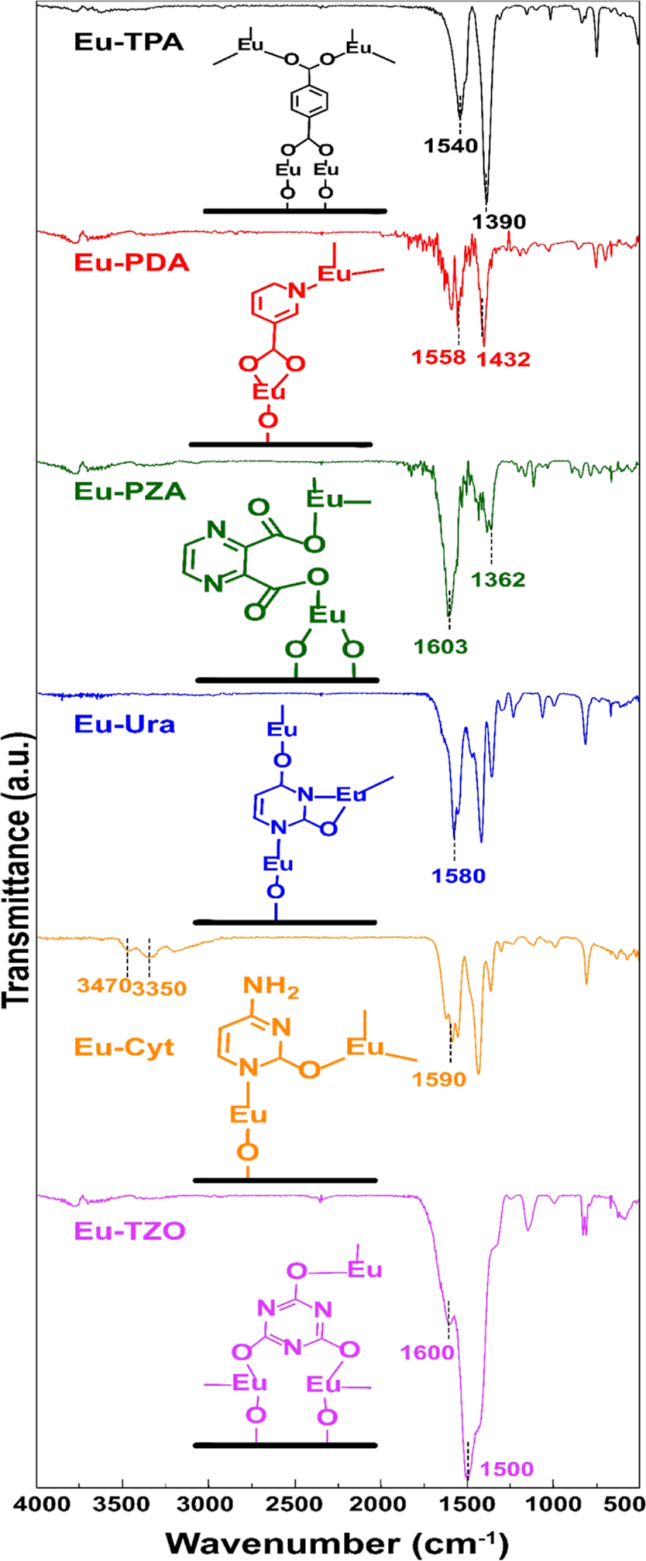
FTIR spectra for the Eu–organic thin films showing possible coordination schemes; the detailed interpretations of the spectral features and comparison to spectra recorded for the precursors are presented in the Supporting Information.

The FTIR spectra recorded for the samples provided us both (i) important evidence of the completeness of the intended chemical reactions leading to the Eu–organic film formation, and (ii) valuable information about the bonding schemes in the resultant hybrid films. The fully interpreted spectra are given for all the films in the Supporting Information together with the corresponding spectra for the precursor powders for reference; in Figure 2 we only emphasize the most indicative spectral features seen for the thin films.

First, for all three thin films grown from different carboxylic acid precursors, the complete reaction of the organic precursor with Eu(thd)_3_ could be confirmed from the absence of any peaks around 1700 cm^–1^ due to unreacted −COOH groups. Also, no indication of the wide peak due to −OH (bending) groups as seen around 2400 cm^–1^ for free TPA, PDA, and PZA molecules (Supporting Information) was seen for any of these thin films. The most characteristic peaks seen around 1550–1600 and 1400 cm^–1^ for these films are due to the asymmetric and symmetric stretching modes of the bonded carboxylate groups. Most importantly, from the separation (Δ) of these peaks, the bonding mode can be deduced, i.e., for unidentate bonding Δ ≫ 200 cm^–1^, bidentate bonding 50 cm^–1^ < Δ < 150 cm^–1^, bridging-type bonding 130 cm^–1^ < Δ < 200 cm^–1^, and ionic bonding Δ ≈ 200 cm^–1^.^21,37^ For the present films, the following Δ values were revealed: Eu–TPA (1540–1390) cm^–1^ = 150 cm^–1^, Eu–PDA (1558–1432) cm^–1^ = 126 cm^–1^, and EuU–PZA (1603–1362) cm^–1^ = 241 cm^–1^, suggesting bridging-type bonding for Eu–TPA, bidentate-type bonding for Eu–PDA, and unidentate-type bonding for Eu–PZA, at least as the majority bonding modes. The bridging-type bonding for Eu–TPA is in line with our previous results for La–TPA and Nd–TPA, and the unidentate bonding in EU–PZA is in line with the observations made for (Y/Yb/Er)-PZA films.^22,32,40^ Since the PDA molecule has only one carboxylic acid group, the continuity of the ideal ALD/MLD type film growth for the Eu–PDA films would require that the nitrogen atom in the pyridine ring also participate in the bonding.

Both Ura and Cyt are pyrimidine (aromatic ring with N atoms at 1 and 3 positions) derivatives. First, a comparison of the FTIR spectra between the Eu–Ura and Eu–Cyt films and the corresponding organic precursors (Ura and Cyt) reveals that the skeletal stretching peak around 1550 cm^–1^ is seen in all four spectra (Supporting Information), confirming that no ring opening occurs upon the thin-film growth.^41,44^ Then, the peaks due to N1H and N3H stretches are seen around 3090 and 2930 cm^–1^ for the Ura precursor (Supporting Information) but are completely missing in the Eu–Ura thin film spectrum, indicating that N1 and N3 are bonded to Eu^3+^ upon the Eu–Ura film growth. Moreover, the peak due to C=O seen at 1710 cm^–1^ for the Ura precursor is shifted to 1580 cm^–1^ for the Eu–Ura film, suggesting that the oxo groups at C2 and C4 also bond to Eu^3+^ in Eu–Ura.

Interestingly, the situation is not completely identical to the Eu–Cyt case. While the Eu^3+^ ions in Eu–Ura are bonded to both ring-nitrogen atoms (N1 and N3) and also to the two oxo groups located at C2 and C4, in Eu–Cyt, the bonding apparently occurs only through N1 (judged by the lack of the peaks at 2670 and 2770 cm due to N1H stretching)^45^ and one oxo group at C2 (judged by the shift of the 1700 cm^–1^ oxo group peak to 1590 cm^–1^). The amino group at C4 remains unbonded in the Eu–Cyt film, judged by the NH_2_ stretching peaks seen at 3470 and 3350 cm^–1^ and the NH_2_ in-plane bending peak at 1625 cm^–1^.

For the TZO precursor, the spectrum shows peaks at 3200 and 3050 cm^–1^ that are attributed to the NH stretch, while the Eu–TZO thin film spectrum lacks such peaks. On the other hand, the strong peak around 1500 cm^–1^ due to the skeletal aromatic ring stretch is clearly seen for the Eu–TZO film (ruling out the possibility of ring opening), leading us to believe that TZO coordinates with Eu as a triol tautomer, while the TZO precursor exists as the trione tautomer. This assumption is confirmed by the absence of the C=O peak at ca. 1770 cm^–1^ and its appearance as the coordinated CO at around 1600 cm^–1^.

Finally, it is interesting to look for correlations between the bonding schemes and the film densities determined for the different Eu–organic thin films from the XRR data. From Table 1, the Eu–TZO film clearly has the highest density (3.56 g/cm^3^). This can be understood by the strong Eu–O bonds from the TZO moiety in three directions. On the other hand, the Eu–PDA has the lowest density (1.58 g/cm^3^), which could be due to the single Eu–O bidentate bonding to the carboxylate group.^46^

### 3.3 Luminescence Properties

**Figure 3:**
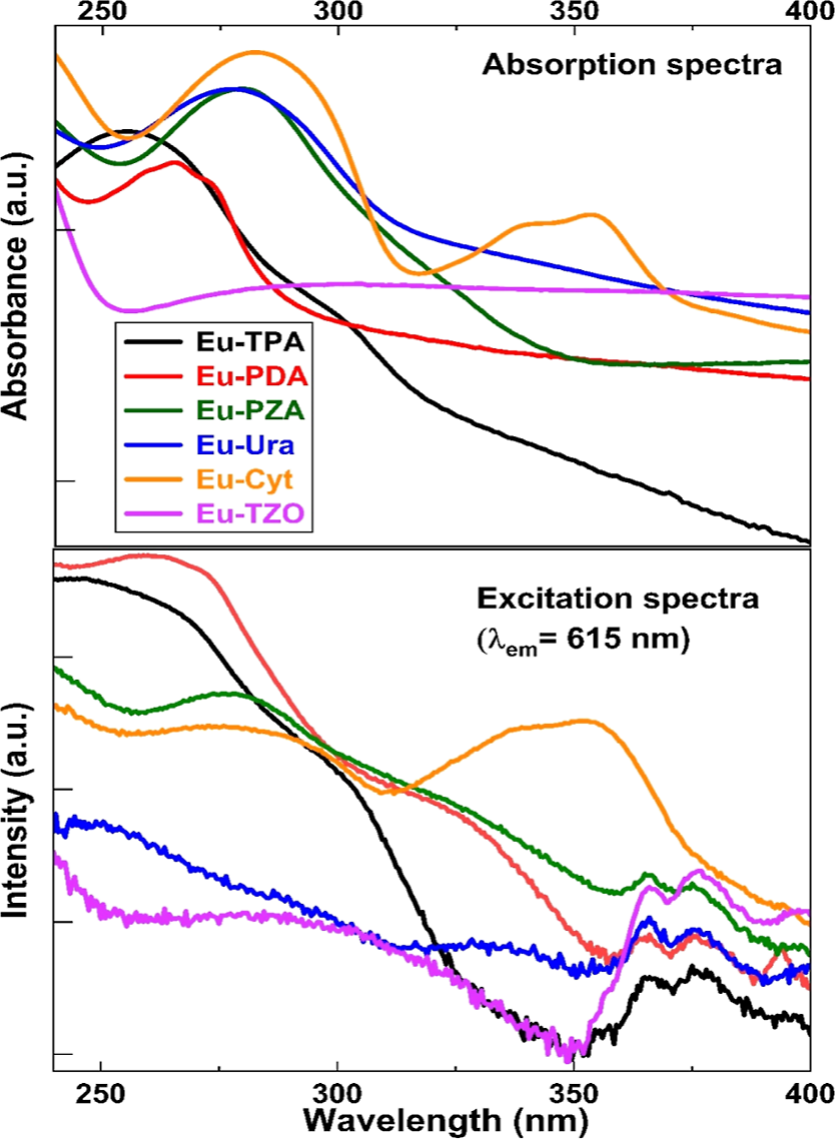
(Top) UV–vis absorption spectra and (bottom) excitation spectra (λ_em_ = 615 nm) for the Eu–organic thin films (film thickness ca. 50 nm). Note: the features between 360 and 400 nm in the excitation spectra are due to the direct scattering of the excitation.

In order to get the first insight into the absorption characteristics of our Eu–organic thin films with different organic components, we measured the UV absorption spectra for the films deposited on quartz glass substrates; these spectra are shown in the upper part of Figure 3. For Eu–TPA, a broad absorption maximum is seen around 250 nm, but the absorption is limited to relatively short wavelengths below 280 nm. Compared to Eu–TPA, for all the nitrogen-containing films except for Eu–TZO, both the absorption maximum and the lower energy tail are shifted to the longer wavelengths. In particular, the Eu–Cyt film shows promising absorption characteristics with two broad and intense absorption peaks covering the wide wavelength ranges of approximately 250–280 and 330–360 nm. We tentatively suggest that the additional −NH_2_ group at C4 could be responsible for the intense second absorption peak centered around 350 nm.

All six Eu–organic thin films showed the red luminescence characteristic of trivalent europium. We thus recorded the excitation spectra for the films by fixing the emission wavelength (λ_em_) at 615 nm, which is the characteristic ^5^D_0_ →^7^F_2_ transition of Eu^3+^; these excitation spectra are shown in the lower part of Figure 3. Figure 3 thus allows the direct comparison between the absorption and excitation spectra, which indeed reveal very similar features. We tentatively interpret the fact that the absorption and excitation spectra are qualitatively very similar for most of the films as an indication that the organic linkers in the Eu–organic films transfer their absorbed energy to the emitting Eu^3+^ ions. The closest similarity between the absorption and excitation spectra is seen for the Eu–Cyt film. This is exciting, as this thin film composition shows a particularly strong excitation capability in the longer wavelength range around 350 nm. Other promising organic components seem to be PDA and PZA with their overall intense excitation spectra in a relatively broad wavelength range, with the tails extending even up to ca. 350 nm.

**Figure 4:**
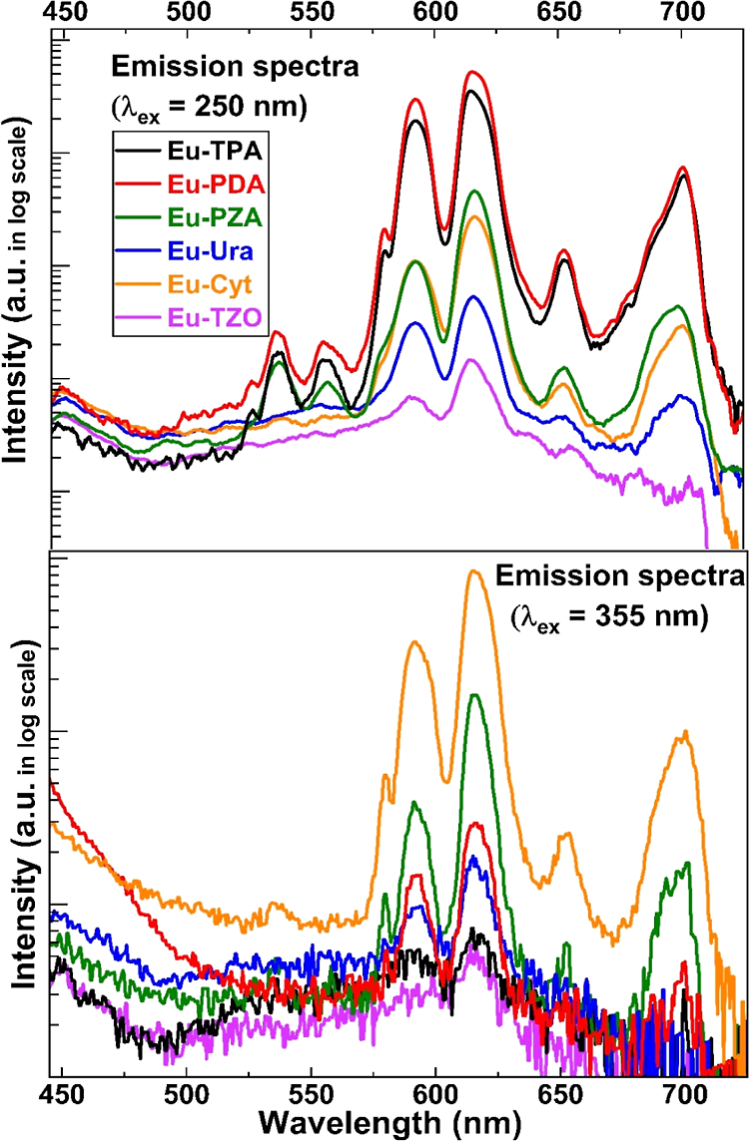
Emission spectra (log-scale) recorded for the Eu–organic thin films with λ_ex_ = 250 nm (top) and with λ_ex_ = 355 nm (bottom).

Figure 4 displays the emission spectra for all the six Eu–organic thin-film samples upon 250 nm (top) and 355 nm (bottom) excitation. Qualitatively, all the spectra are similar, as expected for Eu^3+^ luminescence, but the intensities vary largely (note: intensities in log-scale). With the 250 nm excitation, the luminescence intensity is the highest for the Eu–PDA film, followed by Eu–TPA, while with the 355 nm excitation—in line with the excitation spectra shown in Figure 3—it is the Eu–Cyt film that shows the highest emission intensity. Even though there are no previous data exactly for the same Eu–organic materials as investigated here, the fact that the different organic linkers in Ln–organic materials significantly contribute to the excitation efficiency at different wavelengths and thereby the luminescence intensity is well known in general from the data reported for various Ln–organic bulk materials, as the nature of the organic ring and its substituents definitely have an impact on the absorption wavelength range.^47,48^

A closer look at the emission spectra reveals that the films with the highest overall luminescence intensity (such as Eu–PDA at λ_ex_ = 250 nm excitation, or Eu–Cyt at λ_ex_ = 355 nm excitation) exhibit additional peaks in their emission spectra. These peaks correspond to Eu^3+^ transitions of lower probability and are hence less intense than the major luminescence peaks,^49^ such as the ^5^D_0_ →^7^F_2_ transition peak at λ = 615 nm.^50^ In particular, emissions involving the ^5^D_1_ energy level occur with lower probability and show lower intensity due to the non-radiative relaxation of ^5^D_1_ →^5^D_0_.^51^ In the Supporting Information, the detailed assignments are given for all the seven Eu^3+^ emission peaks. Moreover, the emission spectra for each Eu–organic thin film recorded with an excitation wavelength leading to the highest emission intensity are displayed. The data clearly demonstrate that it is possible—through proper choice of the organic component—to find optimal ALD/MLD-grown thin films for various applications.

**Figure 5:**
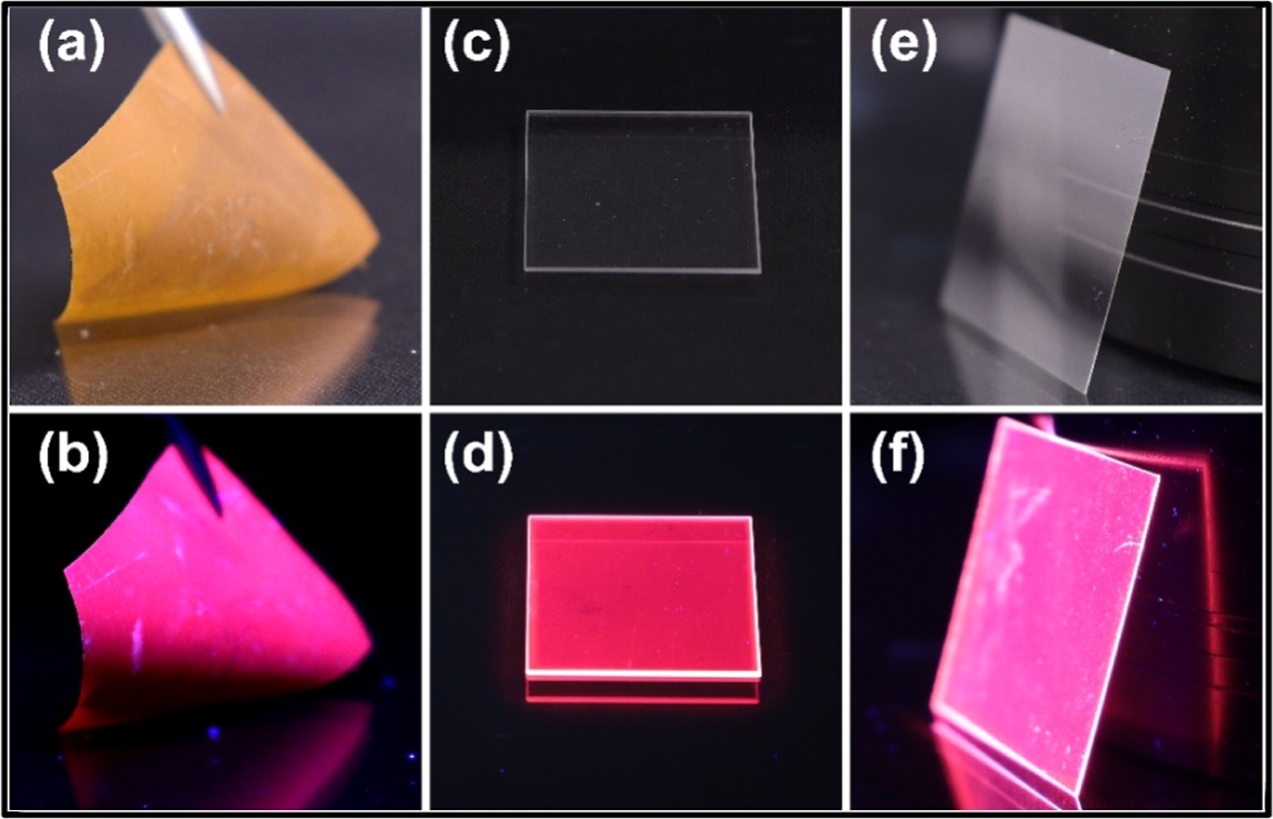
(a) Eu–Cyt on Kapton sheet under room light. (b) Eu–Cyt on Kapton sheet under 302 nm illumination. (c) Eu–PDA on quartz glass under room light. (d) Eu–PDA on quartz glass under 254 nm illumination. (e) Eu–PZA on AF32eco thin glass under room light and (f) under 302 nm illumination.

Finally, we demonstrate the possibility to fabricate these unique ALD/MLD thin film phosphors on various kinds of substrate materials, including flexible and temperature-sensitive ones. Since the deposition temperatures for these thin films ranged between 180–250 °C, we were able to deposit representative film samples on quartz glass, thin AF32eco glass, and polyimide Kapton sheets, listed in the Supporting Information experimental details. In Figure 5, we display photos taken from Eu–Cyt film deposited on a Kapton sheet under room light and 302 nm illumination, Eu–PZA film deposited on AF32eco thin glass under room light and 302 nm illumination, and Eu–PDA film deposited on quartz glass under room light and 254 nm illumination. In particular, the Eu–Cyt film deposited on Kapton demonstrates the mechanical flexibility of our Eu–organic coatings; the red emission remains unaffected even though the substrate was bent in different directions.

The possibility to deposit our Eu–organic films on different substrate materials provided us a way to confirm their reasonably high intrinsic quantum yield (i.e., the ratio between emitted photons to absorbed photons) values as the quantum yield values obtained for (ultra)thin films may significantly depend on the substrate material.^52^ In particular, for the films grown on silicon, the values are strongly reduced due to the fact that silicon absorbs in the visible wavelength range of Eu^3+^ emission. On the other hand, glass does not absorb visible light, but it absorbs some of the excitation UV light. In this work, the quantum yield values were systematically measured for the films grown on silicon; no significant differences were seen regarding the choice of the organic component, but the values remained relatively low (<2.0%) presumably due to the Si absorption. Then, as expected, once the same thin film was grown on non-absorbing substrates, significantly higher quantum yield values could be detected. This was best seen for the Eu–PDA film deposited on quartz glass with a quantum yield value as high as 14.6%.

## 4 Conclusions

We have systematically investigated the role of the organic component on the luminescence characteristics of ALD/MLD-grown Eu–organic thin films. The focus was, in particular, on various nitrogen-containing aromatic organic linker molecules, investigated in comparison to the previously most commonly employed TPA linker. Among the six ALD/MLD processes applied for film fabrication, four were newly developed in this study. All the processes yielded high-quality amorphous Eu–organic thin films with growth rates in the range of 1.56–3.56 Å/cycle. The somewhat different growth rates are perfectly understandable, as the organic precursors differed from each other by their size and the type of reactive groups. The involvement of different reactive groups affects not only the film growth rate but also the way the organic linker molecule is bonded to the Eu^3+^ ions, the latter point then controlling the UV–vis absorption and photoluminescence properties of the films. We investigated the bonding schemes with FTIR spectroscopy and correlated these observations with the film densities determined from XRR patterns.

All six different Eu–organic thin films showed the characteristic red photoluminescence of trivalent europium, but the excitation spectra differed considerably depending on the organic linker molecule. This is of major importance, as it opens exciting opportunities to tune these hybrid materials to match the requirements of different applications. Most importantly, our results demonstrated that we can achieve intense Eu^3+^ luminescence within a broad range of excitation wavelengths even up to visible light excitation. Especially promising results were obtained for the Eu–Cyt films showing a particularly strong and wide excitation peak around 350 nm. On the other hand, for applications requiring shorter wavelength excitation (250–280 nm), Eu–PDA would be a promising candidate. Additionally, the Eu–PDA films (like Eu–PZA) can be deposited at a relatively low temperature of 150–180 °C, thanks to the low sublimation temperature of the PDA precursor, being thus most compatible with temperature-sensitive substrates such as polymers or textiles. These films also possess promising mechanical properties that enable deposition on flexible substrates, such as Kapton sheets, if required. This opens the doors toward new exciting application areas for these flexible luminescent thin films.

## Supporting Information Available

FTIR spectra for the Eu–organic thin films and corresponding precursor powders, interpretations of the FTIR spectral features in these spectra, absorption and excitation spectra for the Eu–organic thin films, emission spectra of Eu–TPA, Eu–PDA, Eu–PZA, Eu–Cyt, Eu–Ura, and Eu–TZO at different excitation wavelengths, observed Eu^3+^ peaks and their interpretations, observed Eu^3+^ emission wavelengths and their respective transitions, photos of representative Eu–organic thin films deposited on various substrates in daylight and under illumination, and XRR patterns for the Eu–organic thin films (ca. 50 nm thick) (PDF)
