# Supplementary material for: Excitation Wavelength Engineering through Organic Linker Choice in Luminescent Atomic/Molecular Layer Deposited Lanthanide–Organic Thin Films
Source: Chem Mater. 2023 Jul 17;35(15):5988–95. doi: 10.1021/acs.chemmater.3c00955 (PMC10413854; doi:10.1021/acs.chemmater.3c00955)
Supplement: Supplementary file 1 — cm3c00955_si_001.pdf [file cm3c00955_si_001.pdf]

Excitation-Wavelength Engineering through Organic Linker Choice in Luminescent Atomic/Molecular Layer Deposited Lanthanide-Organic Thin Films

Amr Ghazy,[a] Mika Lastusaari,[b] and Maarit Karppinen[a*]

[a] Department of Chemistry and Materials Science, Aalto University, FI-00076 Espoo, Finland

[b] Department of Chemistry, University of Turku, FI-20014 Turku, Finland

Email: maarit.karppinen@aalto.fi

Supplementary Information

Figures S1-S5: FTIR spectra for the Eu-organic thin films and corresponding precursor powders.

Tables S1-S5: Interpretations of the FTIR spectral features in these spectra.

Figures S6-S9: Absorption and excitation spectra for the Eu-organic thin films. These spectra were recorded for 50 nm thick samples deposited on quartz glass (absorption) or on silicon (excitation).

Figures S10-S15: Emission spectra of the Eu-organic thin films at different excitation wavelengths.

Figure S16: Eu^{3+} transitions relevant to our Eu-organic thin films.

Tables S6: Emission peaks observed in our Eu-organic thin films and their interpretations.

Figures S17-S23: Photos of representative Eu-organic thin films deposited on various substrates in day light and under illumination.

Figure S24: XRR patterns for the Eu-organic thin films.

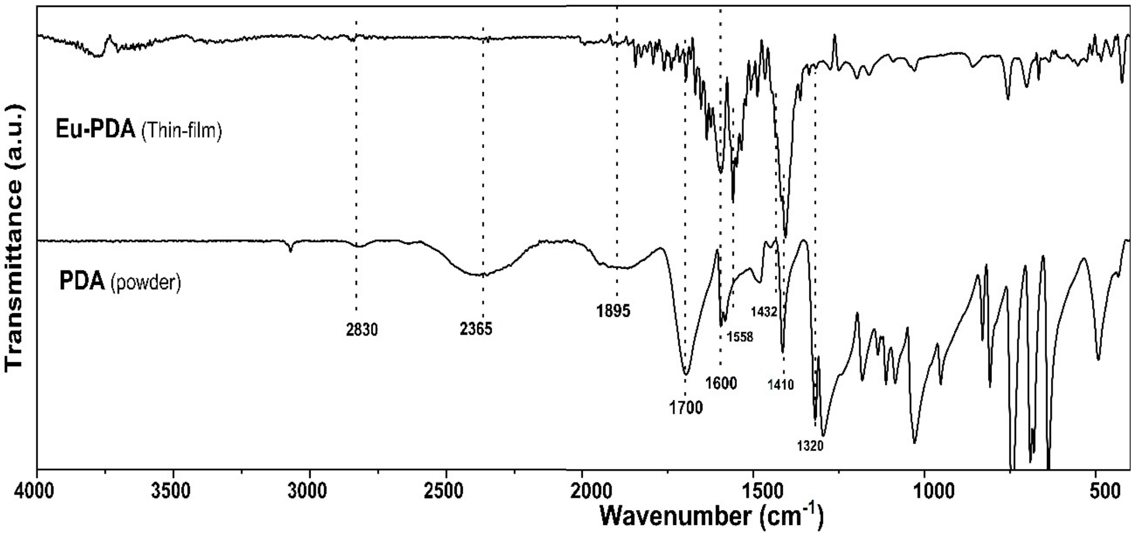

Figure S1. *Comparison between FTIR spectra of PDA precursor and Eu-PDA thin film*

Table S1. *Interpretation of FTIR spectral features*

Mode	PDA	Eu-PDA
OH asymmetric stretch	2830	None
OH symmetric stretch	2365	None
C=O stretching	1895, 1700	1558
C=C stretching	1600	1600
C=N stretching	1410	1410
C=O bending	1432	1320

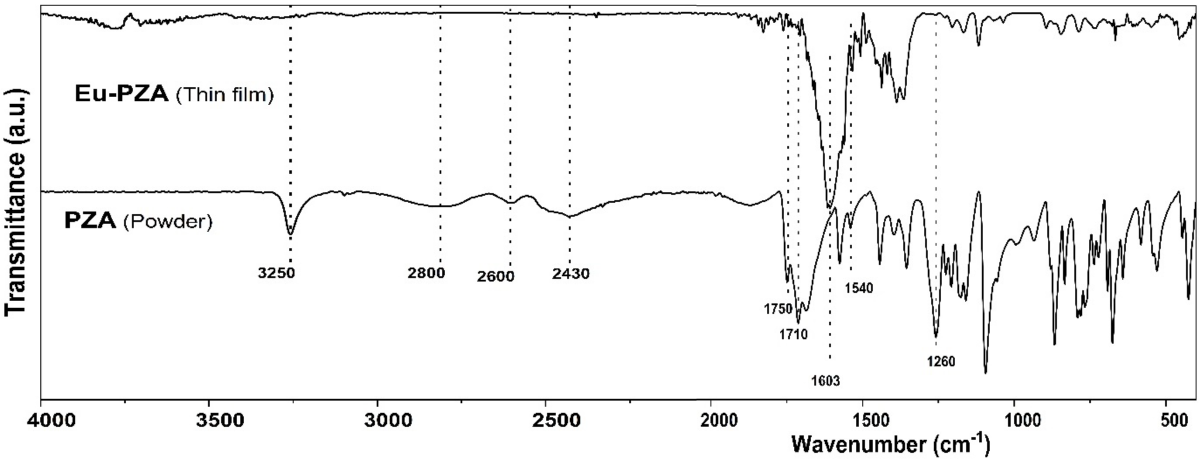

Figure S2. *Comparison between FTIR spectra of PZA precursor and Eu-PZA thin film*

Table S2. *Interpretation of FTIR spectral features*

Mode	PZA	Eu-PZA
OH asymmetric stretch	3250, 2800	None
OH symmetric stretch	2600, 2430	None
C=O stretching	1750, 1710	1603
C-N stretching	1540	1540
C=O bending	1260	1360

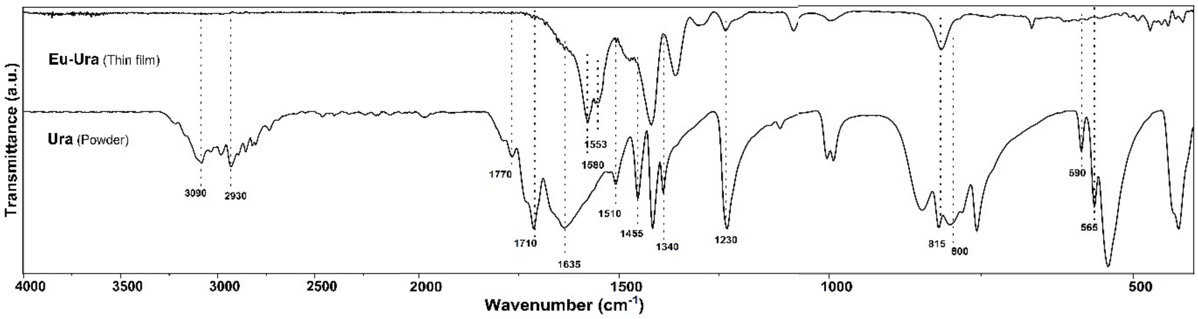

Figure S3. *Comparison between FTIR spectra of Ura precursor and Eu-Ura thin film*

Table S3. *Interpretation of FTIR spectral features*

Mode	Ura	Eu-Ura
N1H asymmetric stretch	3090	None
N3H asymmetric stretch	2930	None
C=O stretching	1770,1710	1580
Skeletal stretch	1560	1553
N1H in-plane bending	1510	None
N3H in-plane bending	1455	None
C=O bending	1340	1420
C=N bending	1230	1235
Skeletal bending	820	815
N3H out of plane bending	850,590	None
N1H out of plane bending	805,565	None

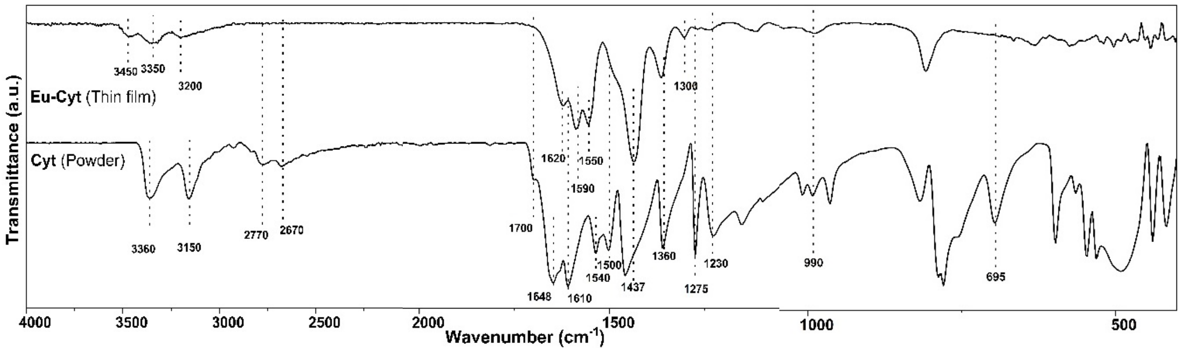

Figure S4. *Comparison between FTIR spectra of Cyt precursor and Eu-Cyt thin film*

Table S4. *Interpretation of FTIR spectral features*

Mode	Cyt	Eu-Cyt
NH_2 asymmetric stretch	3360	3450
NH_2 symmetric stretch	3150	3350
CH stretching	3060	3200
N1H asymmetric stretch	2770	None
N1H symmetric stretch	2670	None
C=O stretching	1700	1590
NH_2 in-plane bending	1648	1620
Skeletal “C3-C4” bending	1540	1550
N1C6 in-plane bending	1500	1500
N3C4 stretching	1437	1437
N3C4 bending	1360	1365
N1H in-plane bending	1275	None
C2N3 in-plane bending	1230	1235
Skeletal “C4-C5” out of plane bending	990	990
N1H out of plane bending	695	None

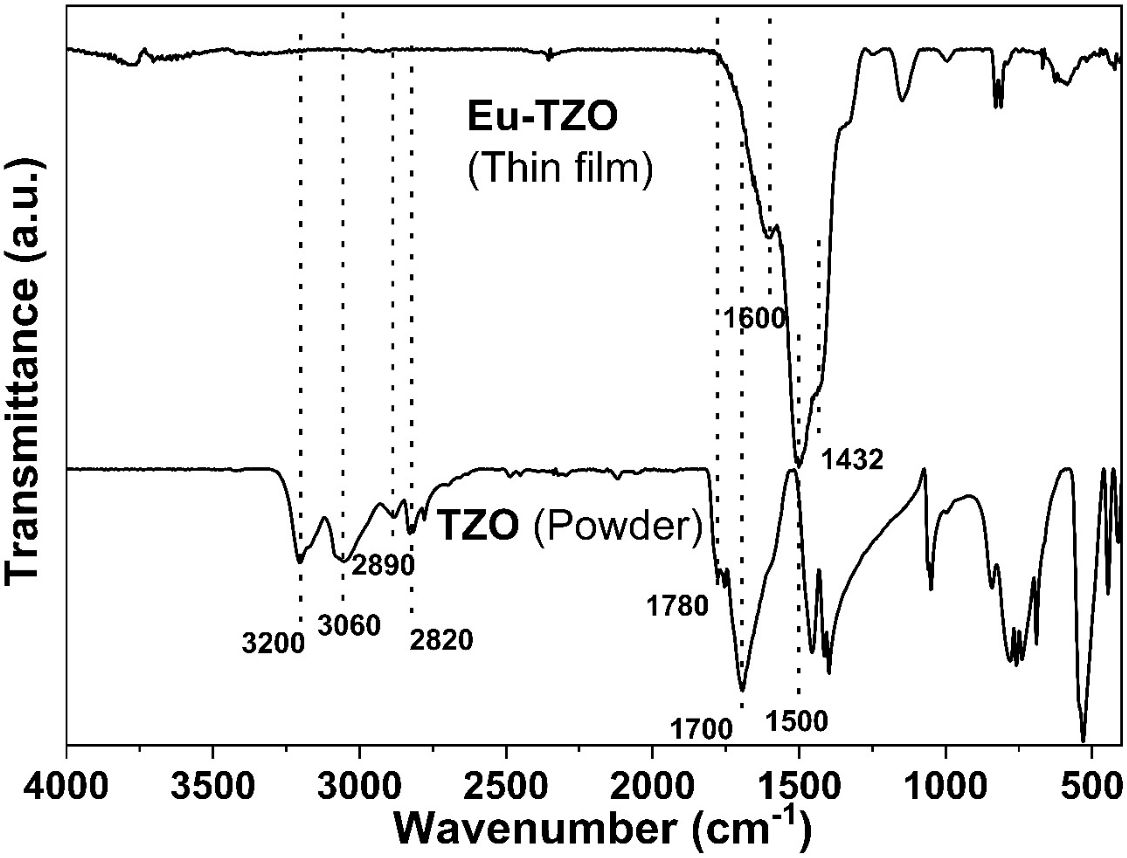

Figure S5. *Comparison between FTIR spectra of TZO precursor and Eu-TZO thin film*

Table S5. *Interpretation of FTIR spectral features*

Mode	TZO	Eu-TZO
NH asymmetric stretch	3200,3060	None
NH symmetric stretch	2890,2820	None
C=O stretching	1780,1700	1600
C-N=C	None	1500
C-NH-C	1460	None
C=O bending	1390	1432

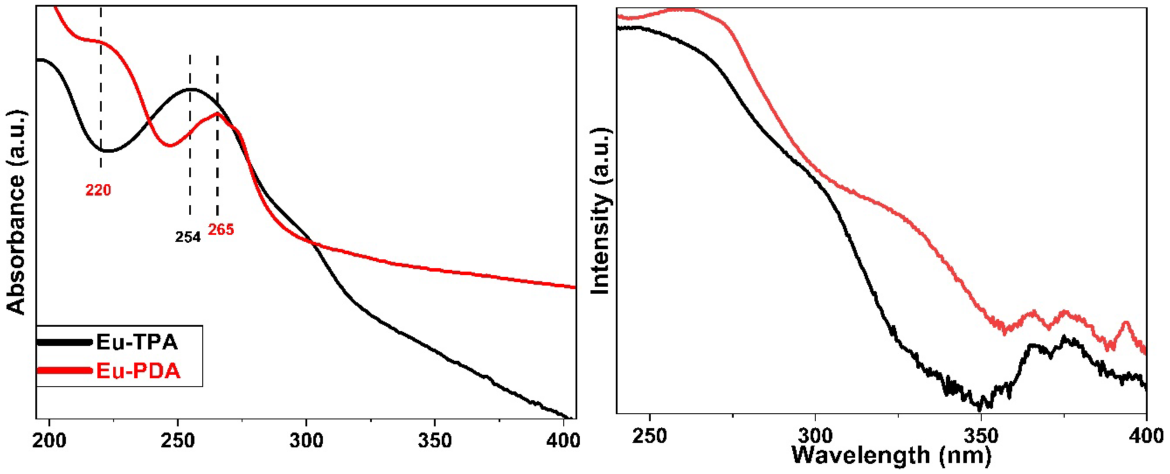

Figure S6. *Comparison between absorption spectra (left) and excitation spectra (right) of Eu-TPA and Eu-PDA*

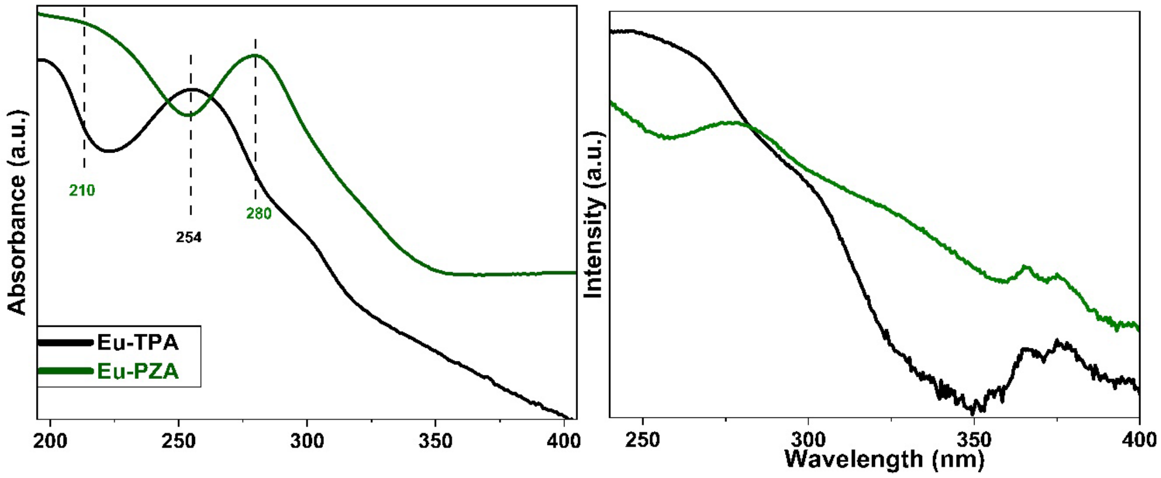

Figure S7. *Comparison between absorption spectra (left) and excitation spectra (right) of Eu-TPA and Eu-PZC*

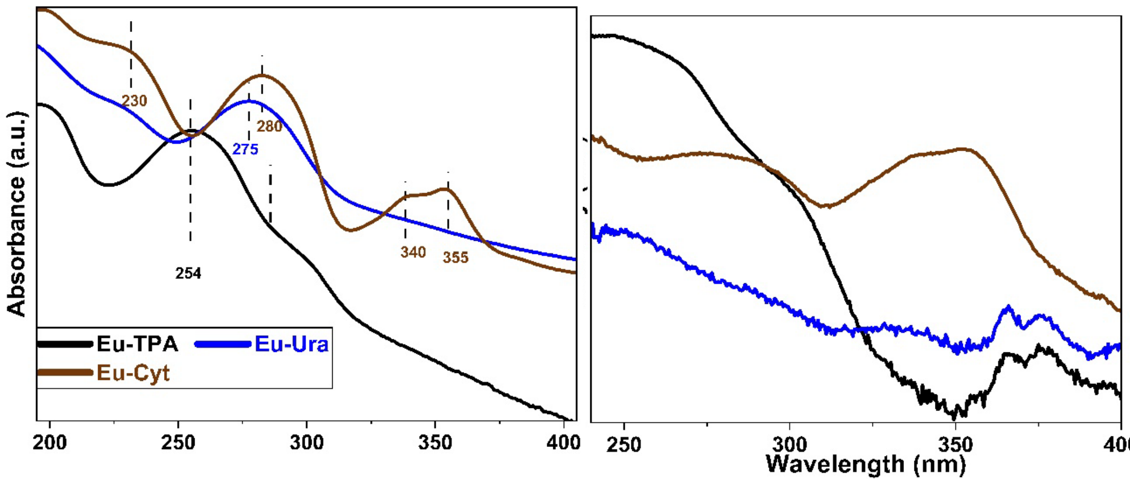

Figure S8. *Comparison between absorption spectra (left) and excitation spectra (right) of Eu-TPA, Eu-Ura and Eu-Cyt*

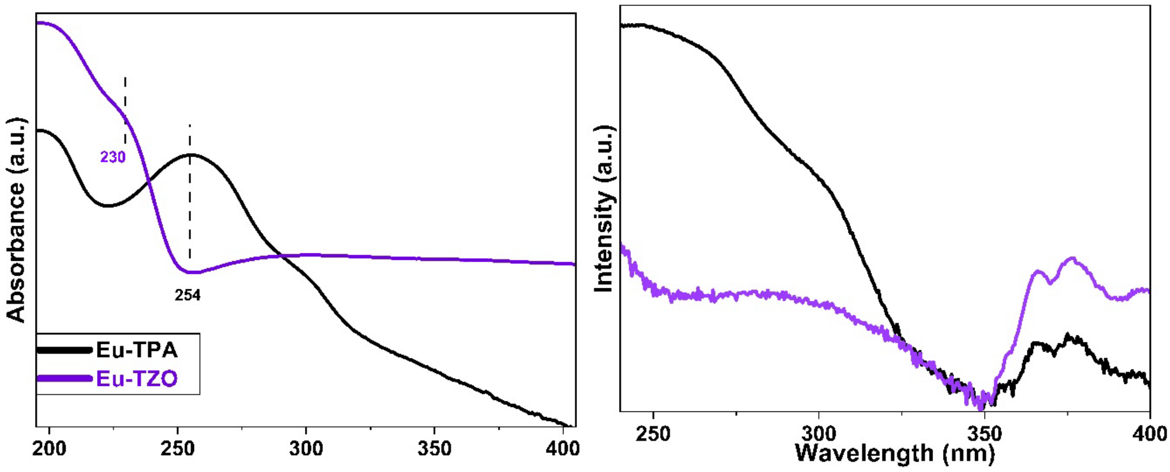

Figure S9. *Comparison between absorbance spectra of Eu-TPA and Eu-CUA*

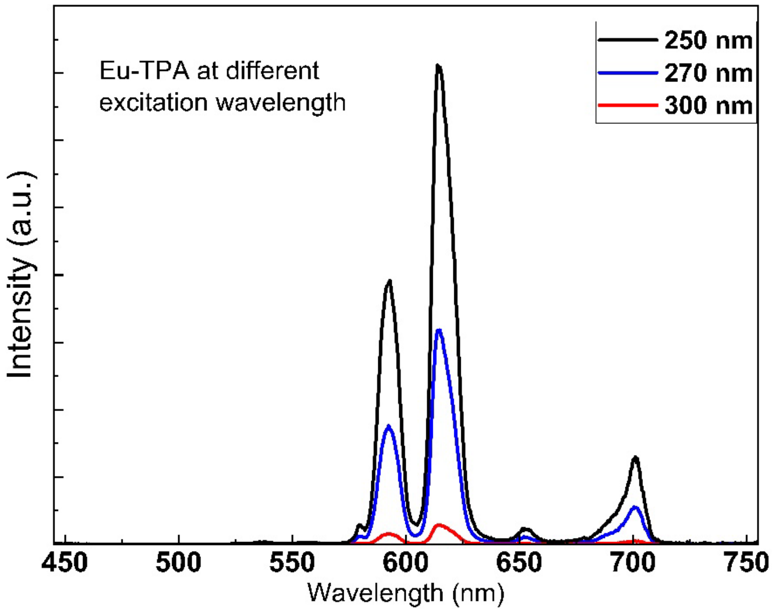

Figure S10. *Emission intensity of 50 nm Eu-TPA thin film on silicon when excited with different wavelengths*

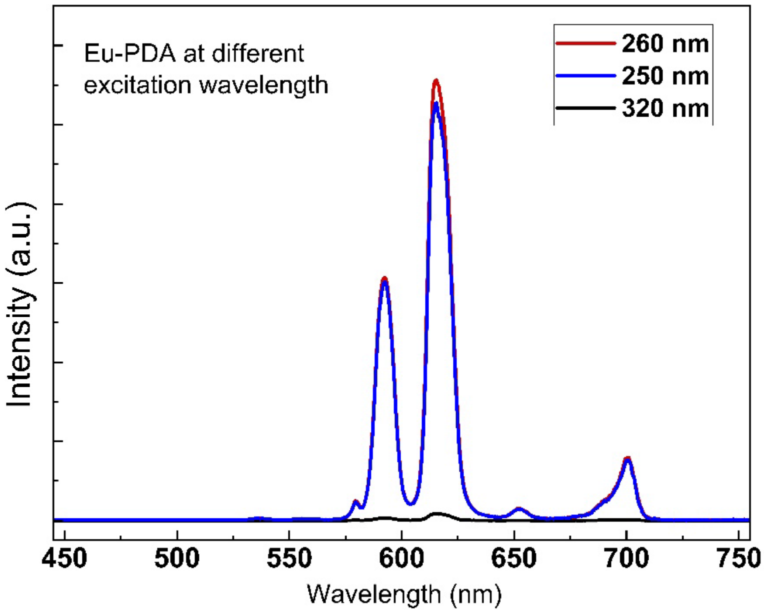

Figure S11. *Emission intensity of 50 nm Eu-PDA thin film on silicon when excited with different wavelengths*

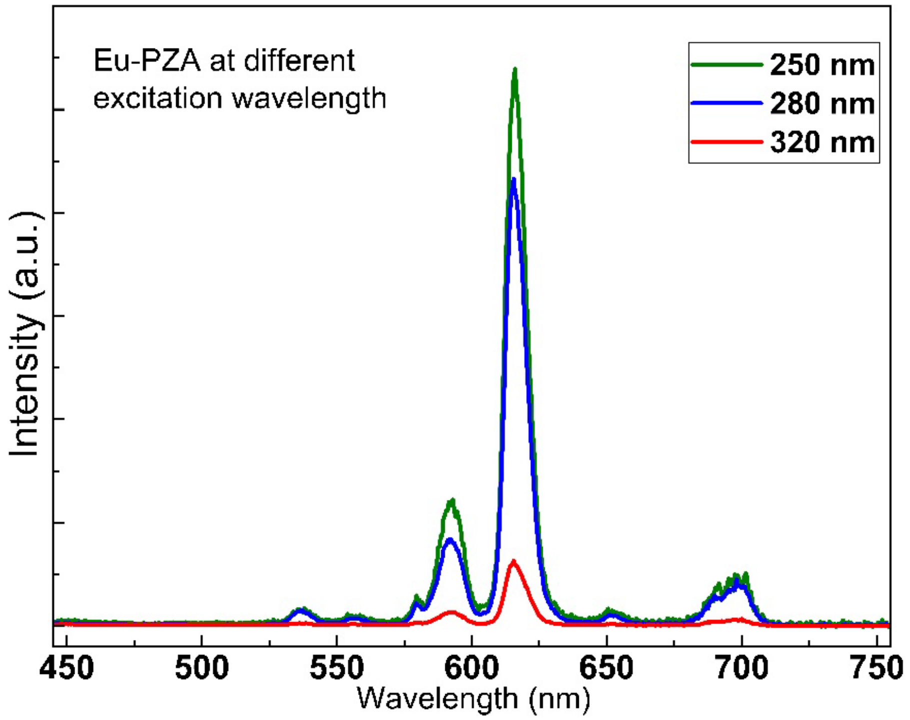

Figure S12. *Emission intensity of 50 nm Eu-PZA thin film on silicon when excited with different wavelengths*

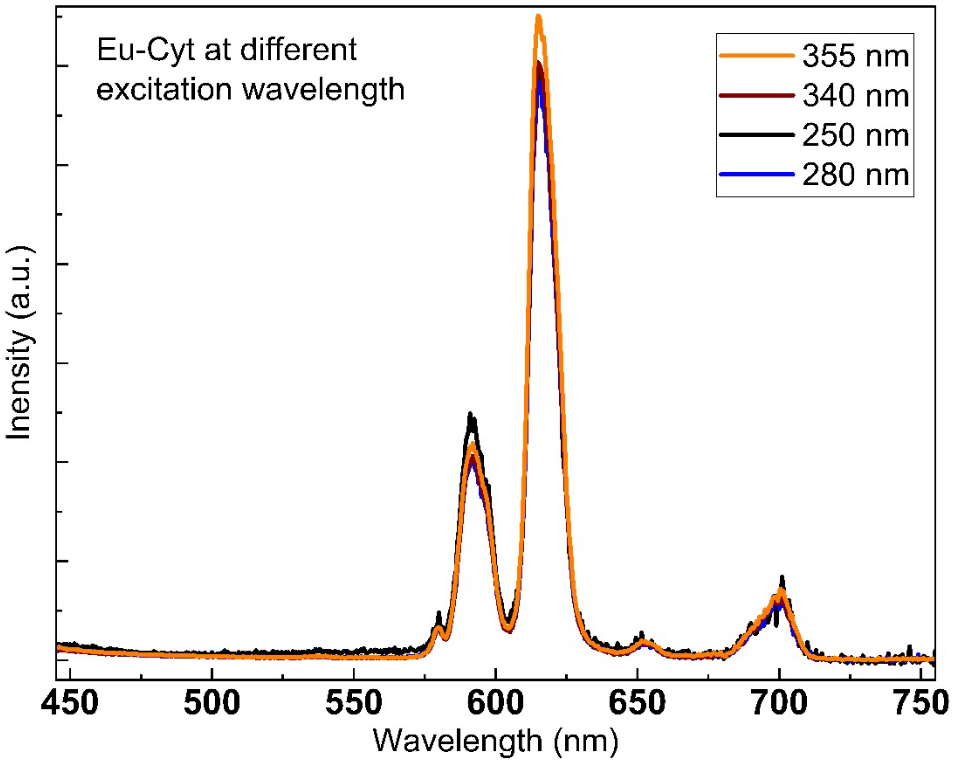

Figure S13. *Emission intensity of 50 nm Eu-Cyt thin film on silicon when excited with different wavelengths*

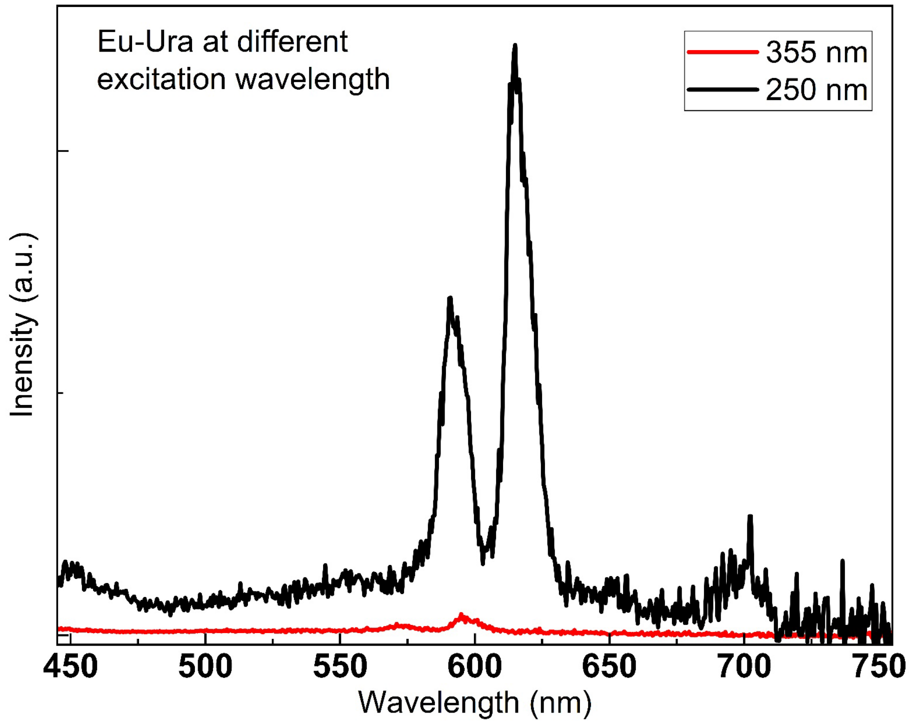

Figure S14. *Emission intensity of 50 nm Eu-Ura thin film on silicon when excited with different wavelengths*

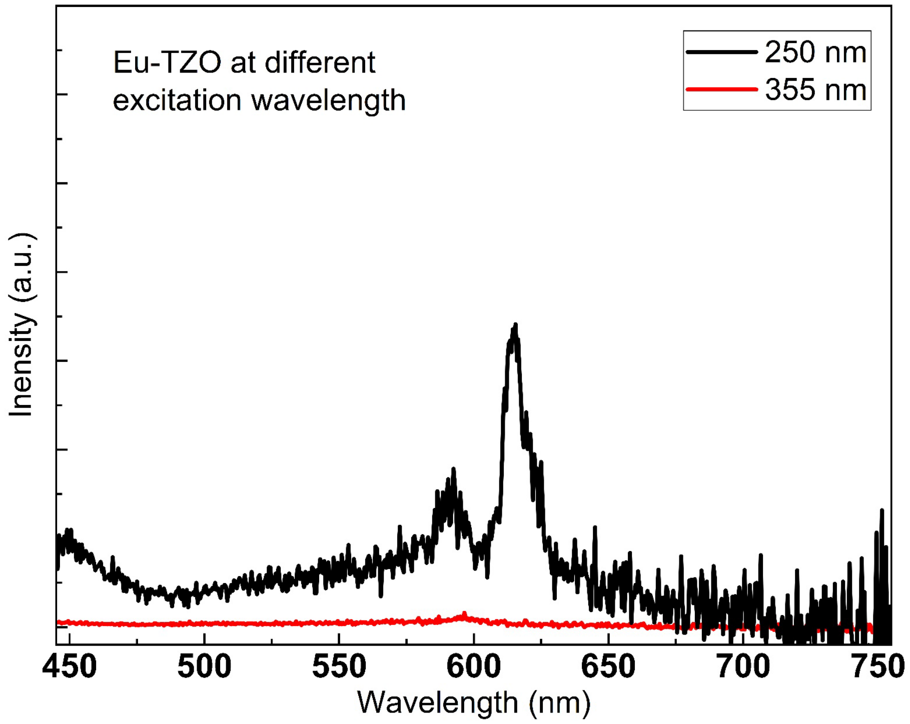

Figure S15. *Emission intensities of Eu-Ura thin film 50 nm sample on silicon when excited with different wavelengths*

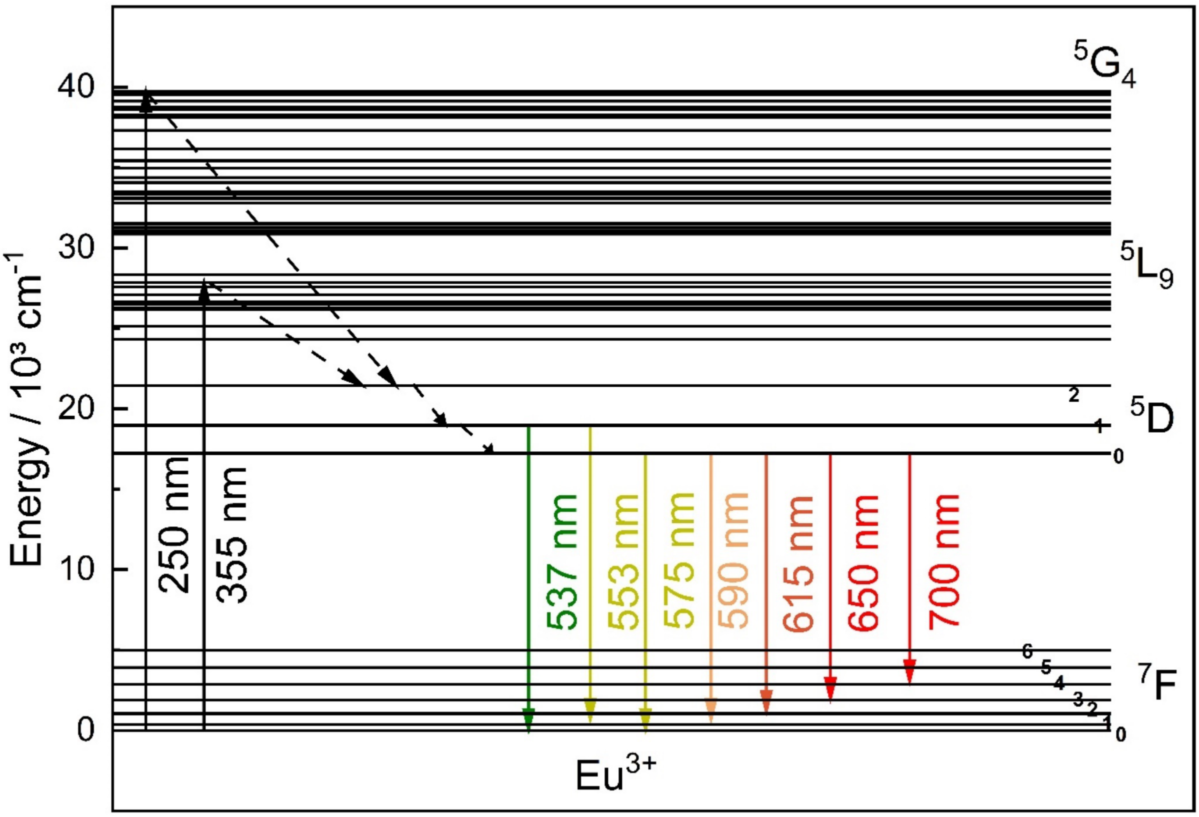

Figure S16. *Eu^{3+} transitions relevant to our Eu-organic thin films*

Table S6. *Emission peaks observed in our Eu-organic thin films and their interpretations*

Emission λ (nm)	Transition
537	$^5D_1 \rightarrow ^7F_1$
553	$^5D_1 \rightarrow ^7F_2$
575	$^5D_0 \rightarrow ^7F_0$
590	$^5D_0 \rightarrow ^7F_1$
615	$^5D_0 \rightarrow ^7F_2$
650	$^5D_0 \rightarrow ^7F_3$
700	$^5D_0 \rightarrow ^7F_4$

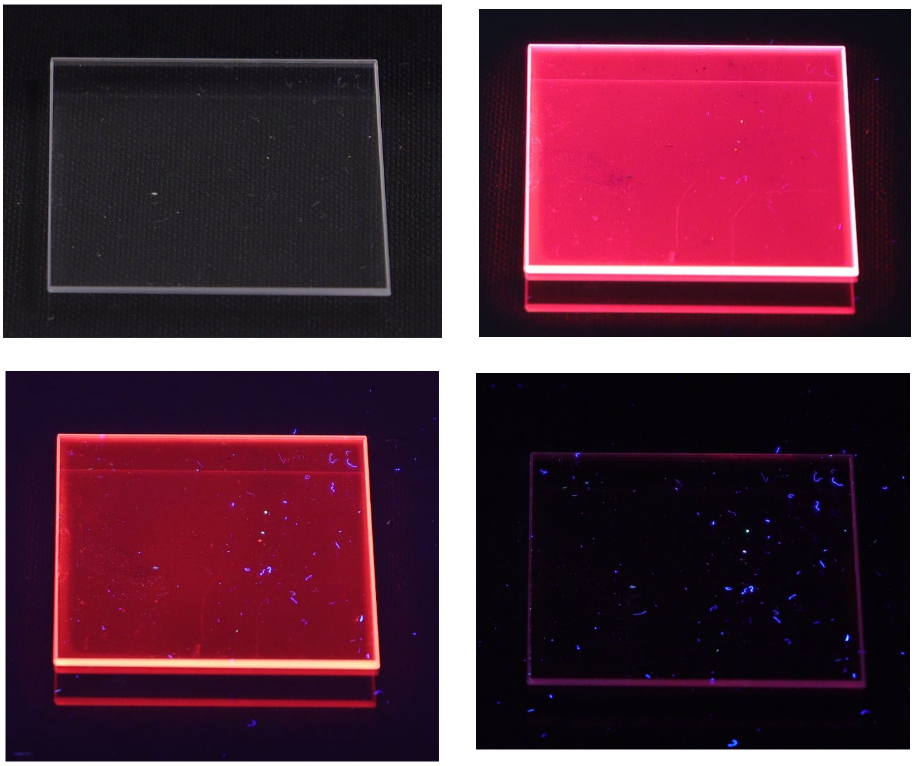

Figure S17. *Eu-PDA on quartz glass (glass substrate thickness = 1 mm), under room light illumination (top left), 254 nm excitation (top right), 302 nm excitation (bottom left), and 365 nm excitation (bottom right). Note: the blue luminescent dots are dust particles*

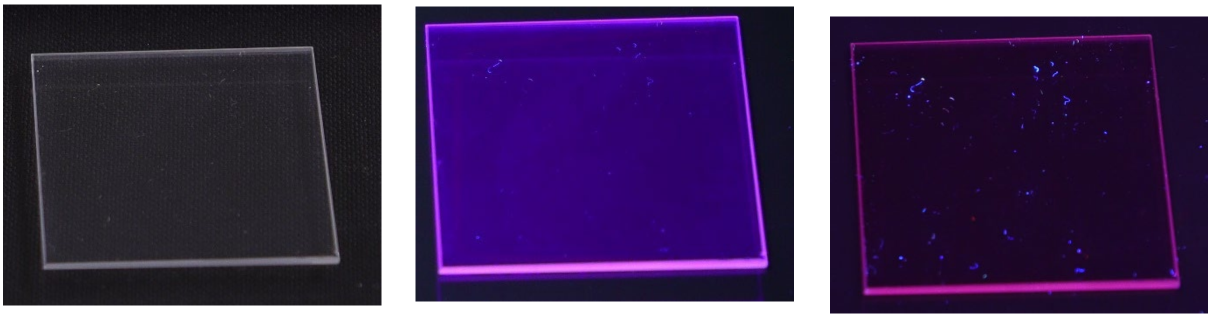

Figure S18. *Eu-Ura on quartz glass (glass substrate thickness = 1 mm. under room light (left), under 254 nm excitation (middle) and under 302 nm excitation (right). Note: the blue luminescent dots are dust particles*

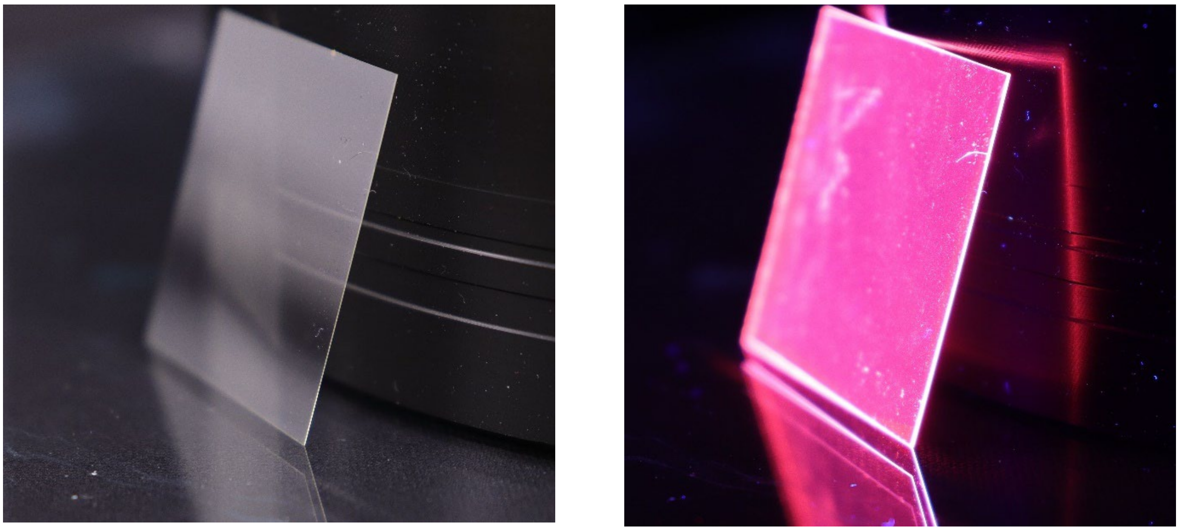

Figure S19. *Eu-PZA on thin AF32 ® eco thin glass (thickness of glass substrate = 0.1 mm) left under room light and right under 302 nm illumination*

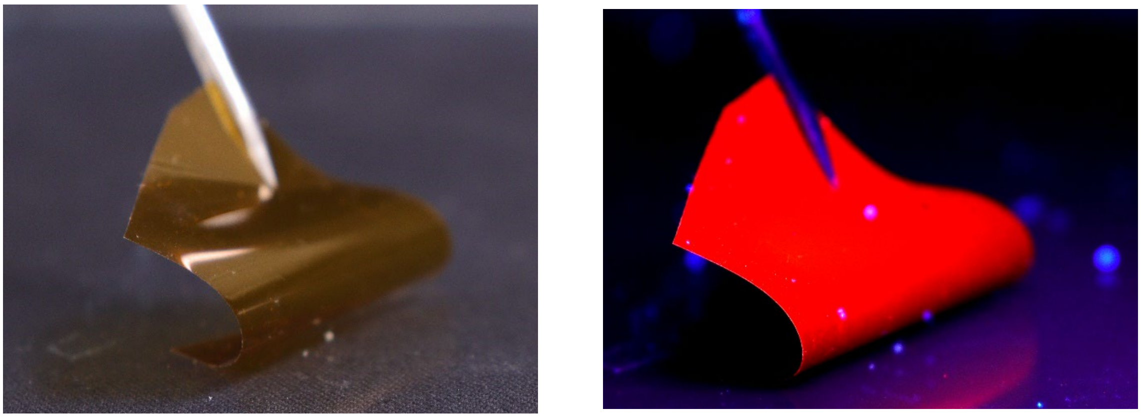

Figure S20. *Eu-PDA on Kapton in room light (left) and under 302 nm illumination (right). Note: the blue luminescent dots are dust particles*

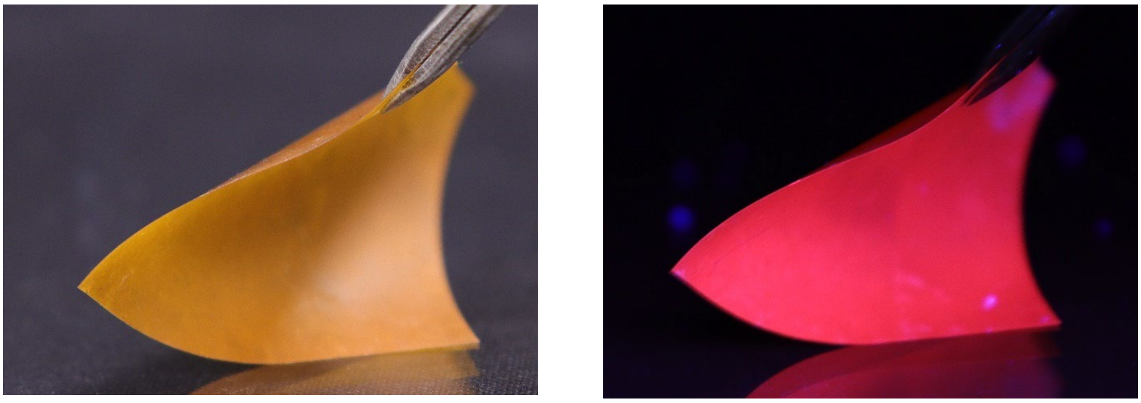

Figure S21. *Eu-Cyt on Kapton in room light (left) and under 302 nm illumination (right). Note: the blue luminescent dots are dust particles*

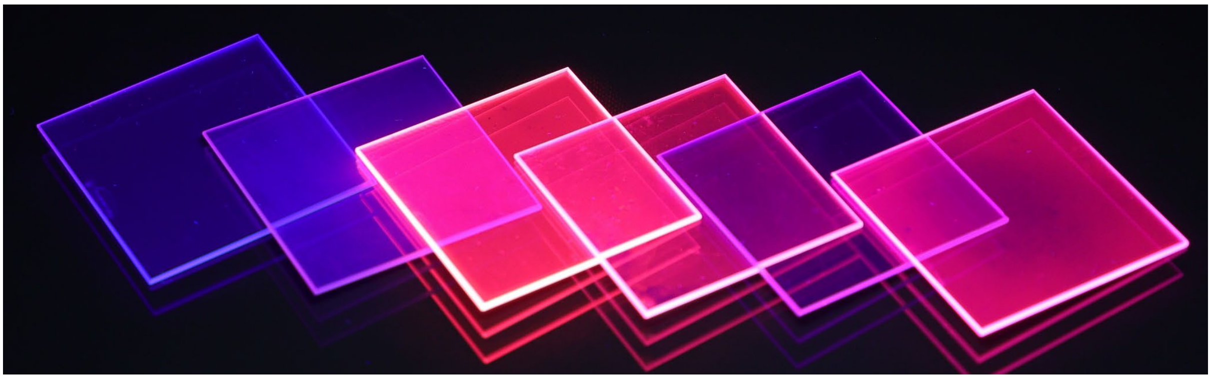

Figure S22. *All six films on glass under 254 nm illumination. From the left: Eu-Ura, Eu-TZO, Eu-PDA, Eu-PZA, Eu-Cyt and Eu-TPA. Eu-PDA gives the strongest emission.*

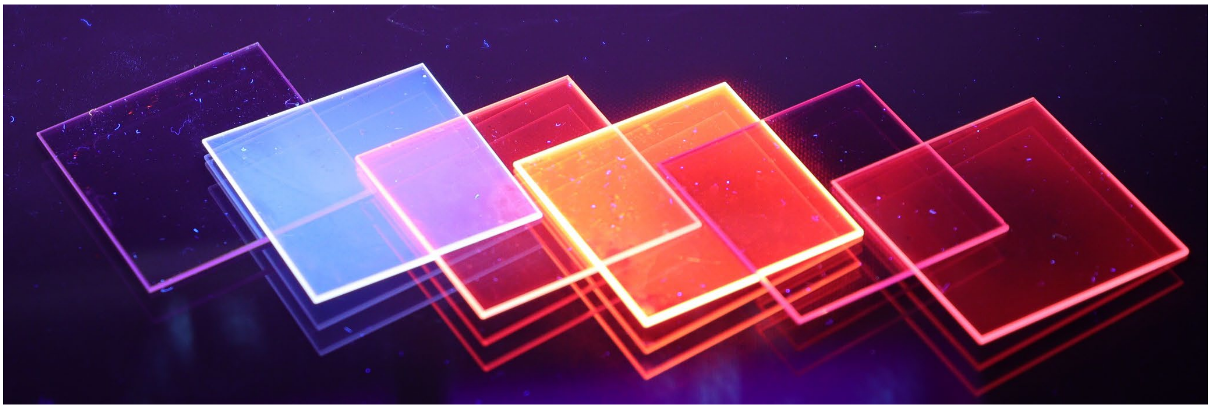

Figure S23. *All six films on glass under 302 nm illumination. From the left: Eu-Ura, Eu-TZO, Eu-PDA, Eu-PZA, Eu-Cyt and Eu-TPA. Eu-PZA gives the strongest emission.*

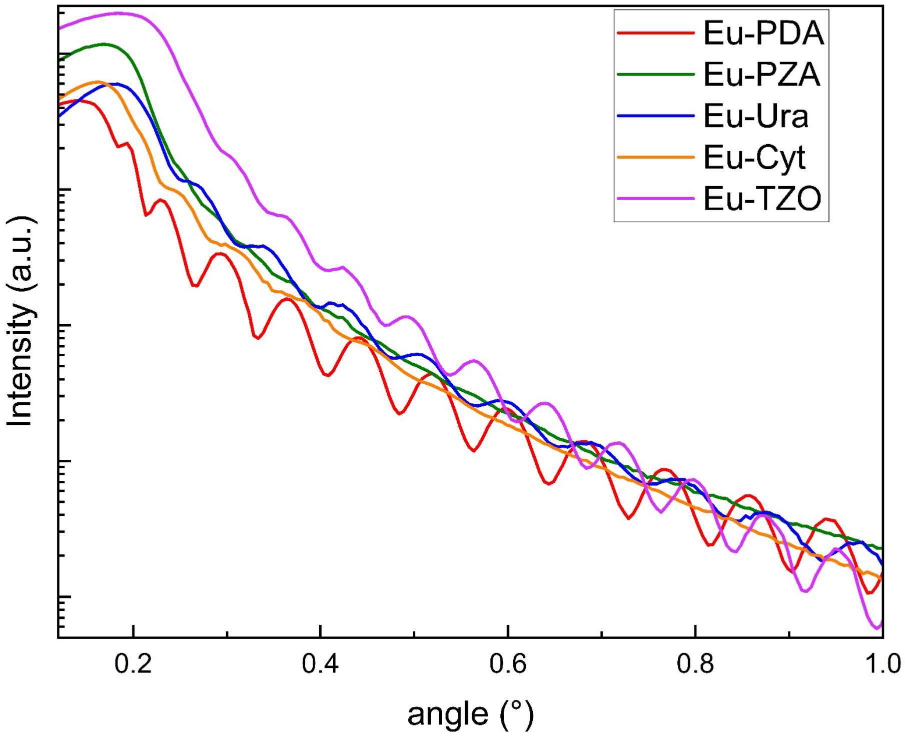

Figure S24. *XRR patterns for ca. 50 nm Eu-organic thin films*
